# Mediation of Systemic Inflammation and Oxidative Stress Markers in the Association of Life’s Crucial 9 with Periodontitis: Evidence from NHANES 2009–2014

**DOI:** 10.3290/j.ohpd.c_2469

**Published:** 2026-01-28

**Authors:** Ruoyao Zhang, Chong Han, Dijia Hu, Qiukai Chen, Jinguo Zheng, Toshinori Okinaga

**Affiliations:** a Ruoyao Zhang PhD Student, Graduate School of Dentistry (Microbiology), Osaka Dental University, Hirakata, Osaka, Japan. Researched, designed, conducted the study, wrote and reviewed the manuscript.; b Chong Han PhD Student, Graduate School of Dentistry (Second Department of Oral and Maxillofacial Surgery), Osaka Dental University, Hirakata, Osaka, Japan. Researched, designed, conducted the study, wrote and reviewed the manuscript.; c Dijia Hu PhD Student, Graduate School of Dentistry (Operative Dentistry), Osaka Dental University, Hirakata, Osaka, Japan. Researched, designed, conducted the study, wrote and reviewed the manuscript.; d Qiukai Chen PhD Student, Department of Microbiology, Osaka Dental University, Hirakata, Osaka, Japan. Researched, designed, conducted the study, wrote and reviewed the manuscript.; e Jinguo Zheng PhD Student, Graduate School of Dentistry (Microbiology), Osaka Dental University, Hirakata , Osaka, Japan. Researched, designed, conducted the study.; f Toshinori Okinaga Professor Department of Microbiology, Osaka Dental University, Hirakata , Osaka, Japan. Researched, designed, conducted the study, reviewed the manuscript.

**Keywords:** depression, Life’s Crucial 9, NHANES, periodontitis, systemic inflammation.

## Abstract

**Purpose:**

To examine the cross-sectional association between LC9 and periodontitis using NHANES 2009-2014 data, while also investigating the roles of systemic inflammation and oxidative stress in this relationship.

**Materials and Methods:**

LC9 was calculated based on the 8 components of LE8 and the depression score assessed by the Patient Health Questionnaire-9. Periodontitis was assessed according to the CDC-AAP definition. Multivariable logistic regression and restricted cubic spline (RCS) analyses were used to explore the relationship between LC9 and periodontitis. Exploratory mediation analyses were performed to examine the roles of systemic inflammation and oxidative stress markers.

**Results:**

A total of 7191 participants were enrolled, and 3540 had periodontitis. In the fully adjusted model, LC9 was inversely associated with the odds of periodontitis (OR per 10-point increase 0.85, 95% CI 0.80-0.90, p < 0.0001). Compared to Q1, participants with LC9 at Q2, Q3, and Q4 had statistically significantly lower periodontitis prevalence (OR 0.78, 0.64, and 0.62, respectively; p for trend = 0.0001). Most LC9 component scores were inversely associated with periodontitis. RCS analysis showed that LC9 was linearly associated with the odds of periodontitis. Exploratory mediation analyses suggested that white blood cell count, neutrophil count, systemic immune-inflammation index, serum albumin, and uric acid may explain 32.53%, 24.05%, 3.64%, 10.70%, and 6.64% of this association, respectively. Stratified analysis showed that age, race, and marital status moderate the relationship between LC9 and periodontitis.

**Conclusion:**

LC9 was linearly and negatively associated with the odds of periodontitis; systemic inflammation and oxidative stress markers may partially explain this association. These findings suggest that LC9 may serve as a valuable, comprehensive tool for assessing the likelihood of developing periodontitis, emphasizing that improving overall cardiovascular and mental health may be associated with lower prevalence of periodontitis.

Periodontitis is a chronic inflammatory and destructive disease that affects the gingiva and periodontal supporting tissues, characterized primarily by periodontal pocket formation, gingival inflammation, alveolar bone resorption, and eventually tooth loss.^1,5^ According to the Global Burden of Disease Study 2021, severe periodontitis is one of the most common oral diseases worldwide.^5^ In the United States, periodontitis affects more than 40% of adults.^33^ In addition, periodontitis is associated with a variety of systemic diseases such as cardiovascular disease (CVD), diabetes, and Alzheimer’s disease.^9,15,49^ Understanding the risk factors for periodontitis is essential for developing novel diagnostics and targeted preventive and therapeutic strategies or biomarkers.^6,16,45,51,54^

Multiple cardiovascular risk factors such as smoking, obesity, and diabetes have been identified as risk factors for periodontitis.^46^ These cardiovascular risk factors may lead to a systemic inflammatory response that affects periodontal tissues and increases the risk of developing periodontitis.^42^ Recently, the American Heart Association (AHA) proposed an updated cardiovascular health (CVH) quantification and monitoring tool known as “Life’s Essential 8” (LE8).^41^ Interestingly, several observational studies have shown that LE8 is inversely associated with the odds of periodontitis in the general population,^11,25,36,39,40,43,50^ suggesting that maintaining ideal CVH may be associated with lower occurrence of periodontitis and serve as a viable preventive strategy.

Accumulating evidence suggests a bidirectional association between psychological health, such as depression, and CVD. Therefore, as an influencing factor for CVH, recent studies have proposed adding psychological health assessment to the existing LE8 framework by proposing a new “Life’s Crucial 9” (LC9).^18,20^ Notably, mounting evidence suggests that psychological health (e.g., depression) may also play a role in the development of periodontitis.^4^ One meta-analysis showed that depression was statistically significantly associated with the prevalence of periodontitis (odds ratio [OR] 1.61, 95% confidence intervals [CI] 1.16-2.23).^37^ More recent cross-sectional analyses suggest that depressive symptoms may be associated with increased odds of periodontitis, although disparate findings exist.^2,26,28^ However, whether the LC9 score is associated with the development of periodontitis still lacks clinical exploration.

In this study, we used nationally representative data from the National Health and Nutrition Examination Survey (NHANES) to explore the cross-sectional association of LC9 with periodontitis in the general population. We aimed to comprehensively investigate whether LC9 is independently associated with periodontitis, reveal the biomarkers that may explain these associations, and explore the potential inequality of these associations in subgroups. These results may help to reveal whether LC9 can be used as a novel CVH assessment framework and raise the possibility that interventions aimed at improving cardiovascular and psychological health might mitigate the burden of periodontitis.

## MATERIALS AND METHODS

### Study Design and Population

NHANES is a nationally representative, population-based, large cross-sectional survey that has been collecting data on participants’ demographics, disease histories, physical examinations, and laboratory tests continuously in two-year cycles since 1999. As a major program of the National Center for Health Statistics (NCHS), NHANES is designed to provide a comprehensive assessment of the health and nutritional status of noninstitutionalized populations in the USA. The study was conducted in accordance with the Declaration of Helsinki; all rounds were approved by the NCHS Ethics Review Board and written informed consent was obtained from participants. NHANES is a de-identified public database, so this study was exempt from local institutional ethics approval.

**Fig 1:**
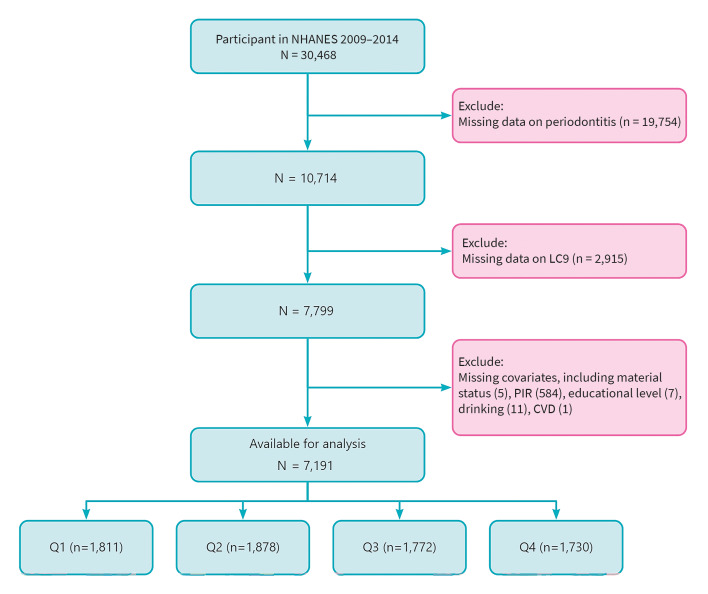
Flowchart of study population selection, NHANES 2009–2014.

This study analyzed publicly available datasets and can be found at https://www.cdc.gov/nchs/nhanes/. The population selection flowchart is presented in Fig 1. First, all 30,468 participants from NHANES 2009-2014 were included. Participants with missing periodontitis diagnosis data (n = 19754) (participants were excluded here primarily because they did not meet the age for oral health-periodontal examination in NHANES, i.e., they were ≥ 30 years old), missing LC9 data (n = 2915), and missing covariates (n = 608) were excluded. Finally, 7191 participants were included.

### Assessment of LC9

The LC9 includes the eight components of the LE8 score with the addition of a depression score.^20^ The LE8 contains 8 health indicators covering health behaviors (diet, physical activity [PA], nicotine exposure, sleep duration) and health factors (body mass index [BMI], blood lipids, blood glucose, and blood pressure).^41^ Each indicator is scored on a scale of 0 to 100. The components of the LE8 score and their assessment methods have been widely reported, and an overview of their assignment details is presented in Table A1. Diet quality was assessed by the Healthy Eating Index-2015 (HEI-2015),^31^ calculated based on 24-hour dietary recall data, and diet quality scores were categorically assigned from 0-100 according to the percentile at which the HEI-2015 was placed. PA, nicotine exposure, and sleep health scores were all collected through standardized questionnaires that assessed participants’ weekly minutes of moderate or vigorous PA participation, smoking/cessation history, and participants’ average hours of sleep per night.^55^ BMI was calculated by dividing weight (kg) by the square of height (m). Blood lipid scores were calculated from plasma non-HDL cholesterol (non-HDL-C, total cholesterol minus HDL-C levels) derived based on laboratory tests. Blood glucose scores were assessed by a combination of fasting blood glucose (FBG), glycosylated hemoglobin A1c, and history of diabetes. Blood pressure scores were assessed by mean (three consecutive) systolic/diastolic blood pressure levels (mmHg) measured at the mobile examination center.

The depression score is evaluated based on the Patient Health Questionnaire-9 (PHQ-9), a validated screening tool for depressive symptoms with high sensitivity and specificity.^48^ An individual’s PHQ-9 score can range from 0 to 27, with higher scores indicating more severe depressive symptoms. The depression score in LC9 was assigned according to the PHQ-9 interval: PHQ-9 at 0-4: 100 points; PHQ-9 at 5-9: 75 points; PHQ-9 at 10-14: 50 points; PHQ-9 at 15-19: 25 points; PHQ-9 at 20-27 points: 0 points.^20^ Ultimately, the LC9 score (taken on a scale of 0-100) is the mean of the 8 components of the LE8 score and the depression score.

### Assessment of Periodontitis

Participants (≥30 years of age) were eligible for periodontal evaluation if they had at least one tooth (excluding the third molar) that did not meet any of the health exclusion criteria. The Oral Health-Periodontal section of the NHANES provides the status of the oral health examination of participants to measure gingival recession (GR) and pocket depth (PD).^39^ The difference between PD and GR was defined as attachment loss (AL). The definition of periodontitis follows the Centers for Disease Control and Prevention-American Academy of Periodontology (CDC-AAP) definition of periodontitis surveillance.^17^ Mild periodontitis was defined as at least 2 interproximal sites with AL ≥ 3 mm and at least 2 interproximal sites with PD ≥ 4 mm (on different teeth), or one site with PD ≥ 5 mm. Moderate periodontitis was defined as at least 2 interproximal sites AL ≥ 4 mm (on different teeth) or at least 2 interproximal sites PD ≥ 5 mm (on different teeth). Severe periodontitis was defined as at least 2 interproximal sites with AL ≥ 6 mm (on different teeth) and at least 1 interproximal site with PD ≥ 5 mm. No periodontitis was defined as the absence of manifestations of mild, moderate, or severe periodontitis. According to previous NHANES studies, patients with mild, moderate, or severe periodontitis were uniformly categorized as periodontitis cases, while those who had no periodontitis comprised the reference group.^11,24,38^

### Covariates

In line with previous related studies,^39,40^ we included multiple key covariates, including age, sex, race, educational level, household income-poverty ratio (PIR), marital status, alcohol consumption, and CVD. Sociodemographic variables were derived from participants’ self-reports on corresponding demographic questionnaires. Alcohol consumption was assessed from the Alcohol Use Questionnaire. Based on previous research, alcohol consumption patterns were categorized as never drinkers, former drinkers, and current drinkers (further categorized as light, moderate, and heavy drinkers).^27^ Never drinkers were defined as having fewer than 12 drinks in their lifetime, and former drinkers were defined as having at least 12 drinks in their lifetime but no drinks in the last year. Current drinkers were categorized according to sex-specific daily alcohol consumption (light drinkers: ≤1 drink/day for women and ≤2 drinks/day for men; moderate drinkers: ≤2 drinks/day for women and ≤3 drinks/day for men or 2–5 days of binge drinking per month; heavy drinkers: ≥3 drinks/day for women and ≥4 drinks/day for men or ≥5 days of binge drinking per month).

### Mediating Variables

Based on previous studies, we included a range of influences that may explain this association.^40^ Systemic inflammation and oxidative stress are important pathogenic mechanisms in periodontitis and may be involved in the crosstalk between CVH/CVD and periodontitis.^14^ Therefore, multiple markers of systemic inflammation and oxidative stress were included. These variables comprised white blood cell (WBC) count, neutrophil count, systemic immune-inflammation index (SII), serum albumin (ALB), C-reactive protein (CRP), gamma-glutamyltransferase (GGT), and uric acid (UA), as well as SII = (platelet count × neutrophil count)/lymphocyte count. Blood cell counts were derived from complete blood count parameters determined by the Beckman Coulter method. Serum ALB, CRP, GGT, and UA were derived from biochemical test data.

### Statistical Analysis

Appropriate sampling weights were incorporated to account for complex survey designs and to yield results that are nationally representative, as recommended by the NHANES analytic protocols. Following the recommendations of the NHANES analytic guidelines, oral health examination sample weights, primary sampling units, and strata variables were incorporated into the 2009-2014 pooled cycle analysis. New six-year sample weights were created by dividing the original two-year weights by three. In baseline, multivariable logistic regression, subgroup, RCS, and sensitivity analyses, the official NHANES analytic guidelines for appropriate weighting were strictly adhered to, ensuring nationally representative results. However, regarding weighting in exploratory mediation analysis, the standard “mediation” package cannot directly handle complex survey designs, so no weighting was applied. Therefore, the results of the mediation analysis should be regarded as exploratory and hypothesis-generating, with precise percentage estimates requiring validation through more advanced methods in prospective studies. Baseline analyses were performed based on LC9 quartiles. Continuous variables were expressed as mean ± standard error and tested by weighted ANOVA, while categorical variables were reported as number (weighted percentage) and analyzed with weighted chi-squared tests. Multivariable logistic regression analysis was used to explore the cross-sectional association of LC9 with periodontitis and to calculate ORs and 95% CIs. Three models were constructed with different levels of adjustment. The crude model did not adjust for any covariates, and model 1 adjusted for age, sex, and race. Model 2 adjusted for educational level, PIR, marital status, alcohol use, and CVD in addition to model 1. A restricted cubic spline (RCS) analysis was used to explore potential nonlinear association between the continuous LC9 score and periodontitis and to select the appropriate number of knots for smooth curve fitting. Whether LC9 indirectly influenced periodontitis through mediating variables was examined in fully adjusted exploratory mediation analyses. The total association of LC9 on periodontitis consisted of a direct association (DA) of LC9 and an indirect association (IA) through mediating variables. The mediating proportion of each individual mediating variable in the total association was calculate. It is important to note that this cross-sectional exploratory mediation analysis cannot infer causality or establish temporal order. The findings should be interpreted as a statistical explanation of the association between variables and serve to generate hypotheses for future longitudinal research. Stratified analyses were applied to explore whether the association of LC9 with periodontitis remained stable in different subgroups of the population and to identify variables that might influence these associations through interaction tests. Multiple sensitivity analyses were conducted. First, we explored whether there were statistically significant associations between LC9 and different severity levels of periodontitis (mild, moderate, or severe periodontitis) and whether linear relationships existed. The baseline characteristic comparisons between the included and excluded populations were supplemented, including differences in key demographic features. Additionally, multicollinearity tests were conducted on the mediating variables. Finally, adjustment was performed for dental flossing history, dental visit frequency, and number of teeth, while not adjusting for CVD in the fully adjusted model (Model 2) to validate the consistency of results. All statistical analyses were performed in R (v4.2.3, R Foundation; Vienna, Austria). A two-sided p<0.05 indicates statistical significance.Publicly available data sets were analyzed in this study. Data for this study are available at https://www.cdc.gov/nchs/nhanes/.

### RESULTS

### Baseline Characteristics

A total of 7191 participants with a mean age of 51.33 years were included. The numbers of participants with no, mild, moderate, and severe periodontitis were 3651, 337, 2490, and 713, respectively. The LC9 quartile distributions were Q1 (<60.6), Q2 (60.6-70.6), Q3 (70.6-79.4), and Q4 (≥79.4). Participants with higher LC9 scores were younger, had higher PIR, and were more likely to be female, non-Hispanic White, non-single, had more than a highschool education, never/light/moderate drinkers, and CVD-free individuals. Participants in higher LC9 quartiles had a statistically significantly lower prevalence of periodontitis (p < 0.0001) (Table 1).

**Table 1:** Baseline analysis according to LC9 quartiles

Variables	Total (n = 7191)	Q1 (n = 1811)	Q2 (n = 1878)	Q3 (n = 1772)	Q4 (n = 1730)	p-value
Age	51.33 ± 0.25	53.57 ± 0.31	52.17 ± 0.39	51.27 ± 0.40	48.98 ± 0.40	<0.0001
PIR	3.26 ± 0.06	2.58 ± 0.07	3.08 ± 0.06	3.35 ± 0.07	3.84 ± 0.07	<0.0001
Diet quality score	44.04 ± 0.56	24.87 ± 0.78	34.52 ± 1.02	46.06 ± 1.03	64.71 ± 0.86	<0.0001
PA score	73.95 ± 0.79	39.55 ± 1.40	69.17 ± 1.41	84.80 ± 1.16	93.80 ± 0.51	<0.0001
Nicotine exposure score	74.11 ± 0.64	49.72 ± 1.32	68.57 ± 1.09	80.60 ± 1.00	91.14 ± 0.62	<0.0001
Sleep health score	83.71 ± 0.41	70.30 ± 0.76	81.09 ± 0.62	87.43 ± 0.59	92.56 ± 0.42	<0.0001
BMI score	59.64 ± 0.59	37.30 ± 1.00	49.53 ± 0.92	62.78 ± 0.81	82.20 ± 0.63	<0.0001
Blood lipids score	60.78 ± 0.50	45.89 ± 0.88	55.04 ± 0.78	61.27 ± 1.01	76.36 ± 0.69	<0.0001
Blood glucose score	84.98 ± 0.40	68.37 ± 1.01	82.88 ± 0.75	88.59 ± 0.56	95.84 ± 0.41	<0.0001
Blood pressure score	68.64 ± 0.58	51.52 ± 0.75	62.60 ± 0.82	69.69 ± 0.88	85.63 ± 0.77	<0.0001
Depression score	92.08 ± 0.34	80.27 ± 0.85	91.78 ± 0.60	95.56 ± 0.45	97.92 ± 0.20	<0.0001
**Sex**						<0.0001
Male	3579 (49.77)	872 (48.09)	1018 (55.21)	952 (53.84)	737 (42.61)	
Female	3612 (50.23)	939 (51.91)	860 (44.79)	820 (46.16)	993 (57.39)	
**Race**						<0.0001
Mexican American	956 (7.09)	246 (7.59)	295 (8.41)	242 (7.65)	173 (5.06)	
Non-Hispanic Black	1401 (9.43)	476 (14.55)	398 (10.91)	332 (8.77)	195 (4.93)	
Non-Hispanic White	3458 (73.02)	817 (68.14)	885 (71.80)	838 (72.89)	918 (77.80)	
Other Hispanic	652 (4.57)	177 (5.40)	156 (4.11)	167 (4.89)	152 (4.10)	
Other Race	724 (5.89)	95 (4.32)	144 (4.77)	193 (5.80)	292 (8.11)	
**Marital Status**						<0.0001
Non-single	4725 (70.43)	1044 (62.07)	1205 (65.99)	1201 (73.04)	1275 (78.19)	
Single	2466 (29.57)	767 (37.93)	673 (34.01)	571 (26.96)	455 (21.81)	
**Education**						<0.0001
Less than high school	533 (3.76)	191 (6.10)	149 (3.95)	113 (3.26)	80 (2.32)	
High school	2456 (29.94)	843 (43.70)	726 (36.89)	569 (28.62)	318 (14.88)	
More than high school	4202 (66.30)	777 (50.20)	1003 (59.16)	1090 (68.12)	1332 (82.80)	
**Drinking alcohol**						<0.0001
Never	879 (9.47)	196 (8.22)	235 (9.91)	217 (9.87)	231 (9.65)	
Former	1251 (14.63)	427 (22.18)	339 (14.97)	304 (14.38)	181 (9.01)	
Light	2664 (40.21)	526 (30.82)	638 (36.56)	681 (41.26)	819 (49.41)	
Moderate	1131 (18.20)	247 (15.97)	301 (17.74)	276 (17.39)	307 (20.97)	
Heavy	1266 (17.49)	415 (22.81)	365 (20.82)	294 (17.10)	192 (10.96)	
**CVD**						<0.0001
No	6585 (92.74)	1539 (85.77)	1719 (92.14)	1660 (94.49)	1667 (96.84)	
Yes	606 (7.26)	272 (14.23)	159 (7.86)	112 (5.51)	63 (3.16)	
**Periodontitis**						<0.0001
No	3651 (59.29)	681 (43.89)	895 (54.38)	937 (61.94)	1138 (72.60)	
Yes	3540 (40.71)	1130 (56.11)	983 (45.62)	835 (38.06)	592 (27.40)	
Continuous variables were expressed as mean ± standard error and tested by weighted ANOVA, categorical variables were reported as number (percentage) and analyzed with weighted chi-squared tests.

### Association of LC9 Score with Periodontitis

**Fig 2:**
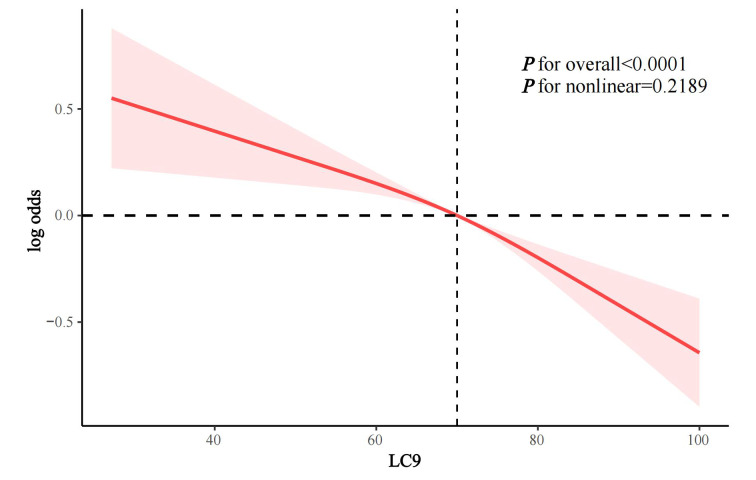
RCS analysis of the association between LC9 and periodontitis.

In the crude model and model 1, LC9 score was statistically significantly negatively associated with the odds of periodontitis (OR 0.70 and 0.73, respectively; both p < 0.0001). In the fully adjusted Model 2, LC9 scores remained negatively associated with periodontitis odds (OR per 10-point increase 0.85, 95% CI 0.80-0.90, p < 0.0001). Compared to Q1, participants in Q2, Q3, and Q4 had a statistically significantly lower prevalence of periodontitis by 22.1% (p = 0.0176), 35.7% (p = 0.0003), and 38.1% (p = 0.0003), respectively, with a statistically significant trend (p for trend = 0.0001) (Table 2). RCS analysis showed a linear association between LC9 score and the odds of periodontitis (p for nonlinear = 0.2189) (Fig 2).

**Table 2:** Association of LC9 with periodontitis in the general US population

	Crude Model	Model 1	Model 2
OR (95%CI)	p-value	OR (95%CI)	p-value	OR (95%CI)	p-value
LC9, every 10 points	0.70 (0.66, 0.73)	<0.0001	0.73 (0.70, 0.78)	<0.0001	0.85 (0.80, 0. 90)	<0.0001
LC9 quartile						
Q1	Ref.		Ref.		Ref.	
Q2	0.66 (0.56, 0.79)	<0.0001	0.65 (0.54, 0.78)	<0.0001	0.78 (0.64, 0.95)	0.0176
Q3	0.47 (0.39, 0.56)	<0.0001	0.47 (0.39, 0.58)	<0.0001	0.64 (0.52, 0.79)	0.0003
Q4	0.30 (0.24, 0.38)	<0.0001	0.37 (0.29, 0.46)	<0.0001	0.62 (0.49, 0.78)	0.0003
p for trend	<0.0001		<0.0001		0.0001	
The crude model did not adjust for any covariates. Model 1 adjusted for age, sex, and race. Model 2 adjusted for education level, PIR, marital status, alcohol use, and CVD in addition to model 1.

### Association of LC9 Component Scores with Periodontitis

**Fig 3:**
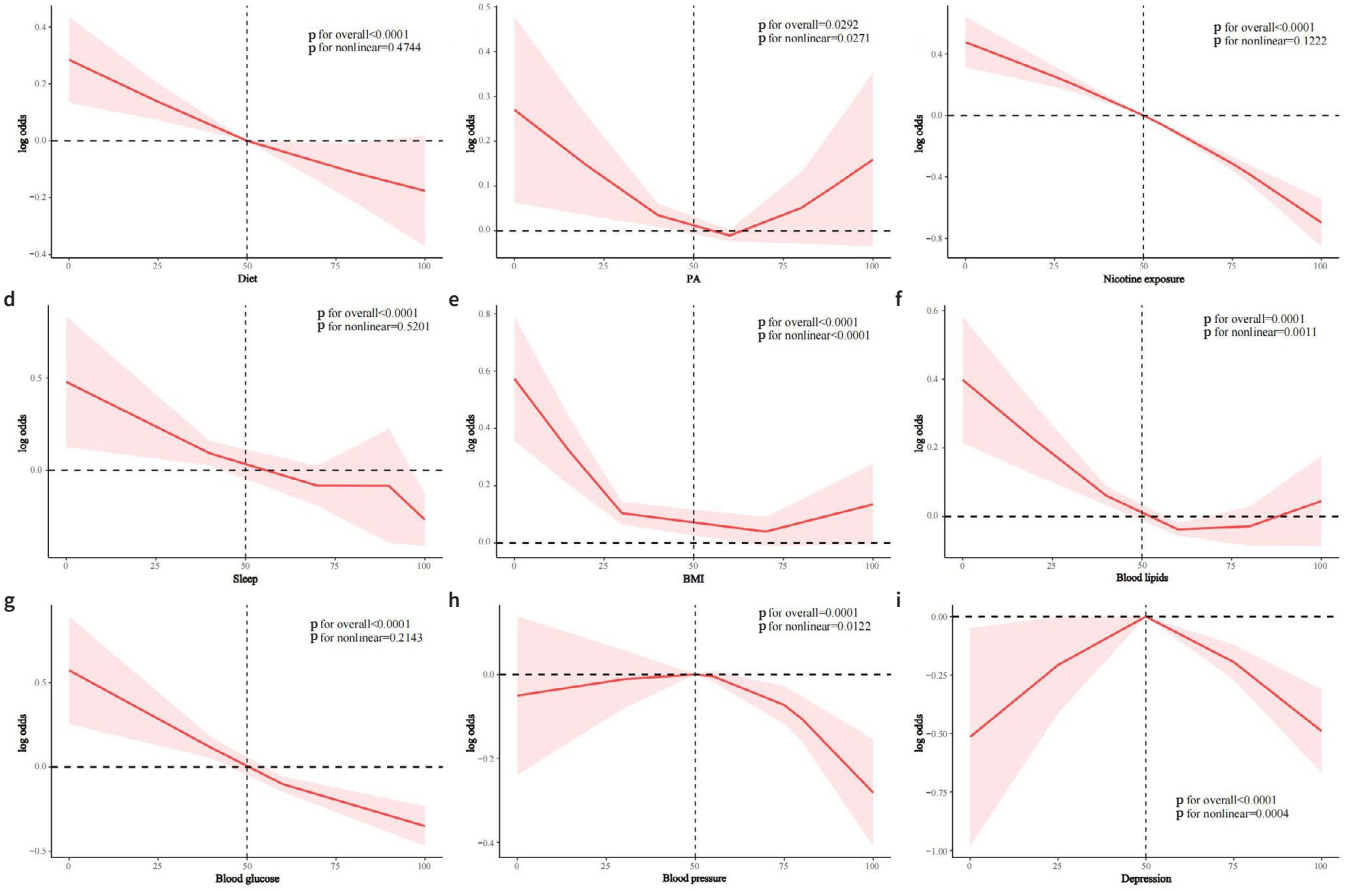
RCS analysis of the association between LC9 component scores and periodontitis. a: diet score; b: PA score; c: nicotine exposure score; d: sleep health score; e: BMI score; f: blood lipids score; g: blood glucose score; h: blood pressure score; i: depression score.

In fully adjusted models, we found that the continuous (per 10-point increase) diet quality score (OR 0.93, 95%CI 0.91-0.94), nicotine exposure score (OR 0.86, 95%CI 0.84-0.87), sleep health score (OR 0.94, 95%CI 0.92-0.96), BMI score (OR 0.98, 95%CI 0.96-1.00), blood lipids score (OR 0.98, 95%CI 0.95-1.00), blood glucose score (OR 0.93, 95%CI 0.90-0.95), blood pressure score (OR 0.97, 95%CI 0.94-0.99), and depression score (OR 0.95, 95%CI 0.90-0.99) were all negatively associated with periodontitis, whereas PA score was not statistically significantly associated (p = 0.1949) (Table 3). RCS analysis showed that diet (p for nonlinear = 0.4744), nicotine exposure (p for nonlinear = 0.1222), sleep health (p for nonlinear = 0.5201), and blood glucose scores (p for nonlinear = 0.2143) were linearly associated with periodontitis, while PA (p for nonlinear = 0.0271), BMI (p for nonlinear < 0.0001), blood lipids (p for nonlinearity = 0.0011), blood pressure (p for nonlinearity = 0.0122), and depression score (p for nonlinearity = 0.0004) were nonlinearly associated with periodontitis (Fig 3).

**Table 3:** Association of LC9 component scores (per 10-point increase) with periodontitis

	Crude Model	Model 1	Model 2
OR (95%CI)	p-value	OR (95%CI)	p-value	OR (95%CI)	p-value
Diet quality score	0.92 (0.90, 0.94)	<0.0001	0.93 (0.91, 0.95)	<0.0001	0.93 (0.91, 0.94)	<0.0001
PA score	0.99 (0.98, 1.01)	0.3110	0.98 (0.97, 1.00)	0.0153	0.99 (0.98, 1.01)	0.1949
Nicotine exposure score	0.86 (0.85, 0.88)	<0.0001	0.87 (0.85, 0.88)	<0.0001	0.86 (0.84, 0.87)	<0.0001
Sleep health score	0.92 (0.90, 0.95)	<0.0001	0.92 (0. 90, 0.94)	<0.0001	0.94 (0.92, 0.96)	<0.0001
BMI score	0.97 (0.95, 0.98)	0.0004	0.97 (0.95, 0.98)	0.0006	0.98 (0.96, 1.00)	0.0168
Blood lipids score	0.97 (0.95, 0.99)	0.0142	0.98 (0.96, 1.00)	0.0687	0.98 (0.95, 1.00)	0.0446
Blood glucose score	0.90 (0.88, 0.92)	<0.0001	0.90 (0.88, 0.93)	<0.0001	0.93 (0.90, 0.95)	<0.0001
Blood pressure score	0.95 (0.92, 0.97)	0.0001	0.96 (0.93, 0.98)	0.0017	0.97 (0.94, 1.00)	0.0185
Depression score	0.96 (0.92, 1.00)	0.0024	0.94 (0. 90, 0.98)	0.0075	0.95 (0.90, 0.99)	0.0156
The crude model did not adjust for any covariates, and model 1 adjusted for age, sex, and race. Model 2 adjusted for education level, PIR, marital status, alcohol use, and CVD in addition to model 1.

### Mediating Role of Systemic Inflammation and Oxidative Stress Indicators

**Fig 4:**
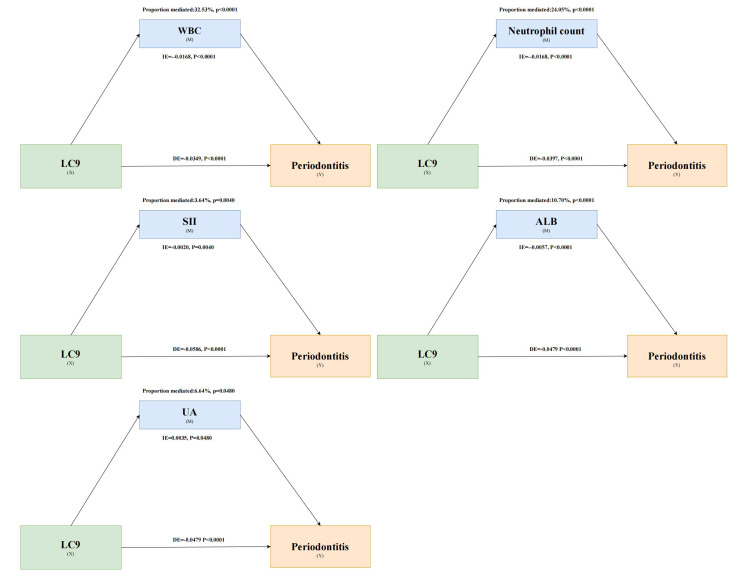
Mediating role of WBC, neutrophil count, SII, ALB, and UA in the association of LC9 and periodontitis.

Exploratory mediation analysis showed that WBC (p < 0.0001 for mediating association), neutrophil count p < 0.0001), SII (p = 0.004), ALB p < 0.0001), and UA (p = 0.048) statistically significantly accounted for this association. The proportions that may potentially be explained by WBC, neutrophil count, SII, ALB, and UA in this association were 32.53%, 24.05%, 3.64%, 10.70%, and 6.64%, respectively (Fig 4). However, CRP (p = 0.118) and GGT (p = 0.25) did not account for a statistically significant association (Tables A2 and A3).

### Stratified Analysis

**Fig 5:**
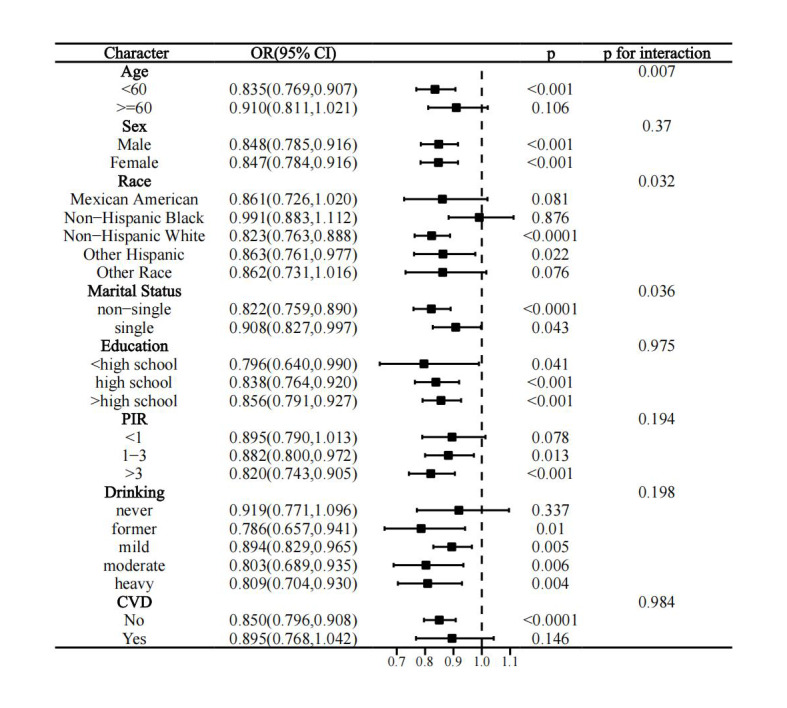
Stratified analysis of the association between LC9 and periodontitis.

Interaction tests indicated that age (p for interaction = 0.007), race (p for interaction = 0.032), and marital status (p for interaction = 0.036) influenced the association of LC9 with periodontitis. This association was more pronounced in the subgroups of <60 years of age, non-Hispanic White, and non-single people (Fig 5).

### Sensitivity Analysis

LC9 was statistically significantly associated with periodontitis across different severity levels (Table A4). The key baseline characteristics of excluded participants were comparable to those of included participants, suggesting that our analysis sample may be representative of the original NHANES sampling population (Table A5). The results of the multicollinearity analysis indicate that the collinearity among these mediating variables is within an acceptable range (Table A6). Adjusting for dental flossing history, mouthwash use, and bone loss around teeth while omitting CVD adjustment from the fully adjusted model did not statistically significantly alter the regression analysis results (Table A7).

## DISCUSSION

This study demonstrates that the newly proposed LC9 score is statistically significantly associated with the odds of periodontitis, manifest as a linear relationship. Our findings reveal that most LC9 components, such as diet, physical activity, and smoking, are inversely correlated with periodontitis. Moreover, systemic inflammation and oxidative stress markers—specifically, WBC count, neutrophil count, SII, ALB, and UA—were found to explain 32.53%, 24.05%, 3.64%, 10.70%, and 6.64% of the association, respectively. Age, race, and marital status were found to statistically significantly moderate this association, with the association being more pronounced in individuals under 60 years old, non-Hispanic Whites, and non-single participants.

Gaffey et al^18^ first proposed the addition of psychological health assessment to the LE8 framework to form the LC9 in 2024. To the best of our knowledge, this study is the first to explore the association of LC9 with periodontitis, utilizing data from a large representative sample. Previous studies examining the association between LE8 and periodontitis found consistent results, showing that higher LE8 scores are linked to a lower prevalence of periodontitis.^11,25,36,39,40,43,50^ Our study builds on this body of research, confirming that higher LC9 scores are also associated with lower odds of periodontitis, further supporting the idea that CVH is crucial for oral health. Notably, this association was stronger in younger, non-Hispanic White and non-single populations, suggesting that targeted interventions for these subgroups may be particularly beneficial. The integration of depression assessment into the LE8 framework to form LC9 is grounded in the well-established bidirectional relationship between psychological and cardiovascular fitness. This approach aims to provide a more comprehensive evaluation of individual health status, reflecting the interplay between physical and mental dimensions. While this composite measure presents conceptual challenges in equating psychological and biological metrics, it represents an evolving paradigm for holistic health assessment that warrants empirical exploration. The authors acknowledge the conceptual complexity of combining the depression score with the LE8 biomedical indicators into a single average score. In this study, the concept and assessment of LC9 are based on previously published high-quality research, ensuring comparability and reproducibility. However, it is also recognized that the consistency of assessment methods between the depression score and other LE8 indicators remains an area requiring further exploration and investigation. The current LC9 assessment framework remains theoretical in nature, and our research is positioned as “exploratory” and “hypothesis-generating”.

The mechanisms linking LC9 to periodontitis remain poorly understood experimentally, and it is hypothesized that systemic inflammation may be involved in this association. The observed association coupled with the explanatory role of inflammatory markers lends support to a plausible model in which better cardiovascular and psychological health contributes to a less pro-inflammatory systemic state, which in turn may mitigate the inflammatory processes in the periodontal tissues. Chronic systemic inflammation has long been recognized as a key factor in the development and progression of periodontitis.^7,19^ In addition, systemic inflammation and oxidative stress are closely related.^56^ Our exploratory mediation analysis suggests that LC9 may contribute to higher odds of periodontitis through systemic inflammation and oxidative stress markers; therefore, it is plausible that systemic inflammation may be a key crosstalk factor between CVH and periodontitis. These findings provide new insights into the lower prevalence of periodontitis given higher CVH and offer new perspectives and strategies for the prevention and treatment of periodontitis. It should be noted that the exploratory mediation analysis in this study can only indicate the presence of statistical associations. It aims to generate hypotheses about the complex relationships among LC9, systemic inflammation, and periodontitis, rather than test a specific causal model.

Depression may contribute to poor oral hygiene and increased susceptibility to periodontal disease, as individuals with depression are more likely to adopt unhealthy behaviors, such as smoking and poor diet.^52^ Furthermore, depression is associated with a chronic inflammatory state, which may exacerbate periodontal inflammation and immune dysfunction.^13,23^ Animal experiments have also provided relevant evidence. Use of the tricyclic antidepressant tianeptine, which has anti-inflammatory effects, reduced the severity of periodontitis induced by ligation in an olfactory bulbectomy rat model.^23^ Our findings similarly suggest that the depression score assessed by the PHQ-9 was statistically significantly associated with periodontitis. While many studies have explored the link between depression and periodontitis,^2,4,28,32,37,47^ the present study systematically assessed depression as part of the LC9 framework and examined its exploratory mediation association. Our findings suggest that depression, as assessed by the PHQ-9, significantly contributes to the association between LC9 and periodontitis. However, the relationship between depression and periodontitis appears to be complex and may vary depending on the severity of depression,^3,8,29^ which may explain some of the inconsistencies found in previous studies. Future prospective cohort studies will be essential to clarify the role of depression in the development of periodontitis and to explore the nonlinear effects that may exist.

The current study has several strengths. It is a multiethnic, population-based study based on NHANES, making the results potentially representative of the US population. The large representative sample allows for potential extrapolation and reliability of the findings to better reflect the reality of the US population. This is the first time that the association of the newly proposed LC9 score with periodontitis has been investigate, and this comprehensive assessment provides new perspectives on risk factors and prevention strategies for periodontitis.

The periodontitis classification algorithm and LE8 scoring criteria used in the study were also rigorously defined to ensure the accuracy and consistency of the data. However, this study has some noteworthy limitations. Being cross-sectional in design, the temporal order of associations and causal inferences could not be determined. Indeed, the primary limitation of this study lies in its cross-sectional design, which precludes any causal inferences. The observed associations and proposed mediating role of inflammatory markers may be confounded by reverse causality. For instance, pre-existing periodontitis, as a chronic inflammatory condition, may itself elevate systemic inflammation levels and adversely affect mental health, thereby influencing LC9 scores. Prospective cohort studies are needed to validate temporal sequences and causal pathways. Assessment of some components of LC9 relied on self-report and may be affected by recall bias. Although the study adjusted for multiple covariates, there may have been residual confounders (e.g., genetic susceptibility and dental factors). Such unmeasured factors in NHANES need to be addressed in future studies. Another important limitation was that the assessment of psychological health relied only on depressive symptoms assessed by the PHQ-9, which was unable to comprehensively measure the multidimensionality and complexity of psychological health (e.g., the potential role of anxiety, loneliness, chronic stress, etc). Given the limitations of the NHANES data, more comprehensive mental health factors need to be included in the future to adequately reflect LC9. Overall, these findings suggest that maintaining higher LC9 scores is associated with a lower prevalence of periodontitis, and our findings raise the possibility that interventions aimed at improving cardiovascular and psychological health might also have beneficial potential on periodontal outcomes, although longitudinal studies are needed for validation.

## CONCLUSIONS

LC9 was inversely correlated with the odds of periodontitis in the US general population and exhibited a linear association. Most LC9 component scores were negatively associated with periodontitis. WBC, neutrophil count, SII, ALB, and UA may provide a potential explanation. This association was more pronounced in groups <60 years of age, non-Hispanic White, and non-single populations. These findings suggested that maintaining higher LC9 was associated with lower odds of periodontitis and support the possibility that interventions aimed at improving cardiovascular and psychological health might mitigate the burden of periodontitis.

## ACKNOWLEDGMENTS

We are grateful to all of the researchers and participants in NHANES.

## Appendix

p

p

p

p

p

p

p

p

p

p

p

p

p

p

p

p

p

p

p

p

**Table A1:** Definition and scoring approach for the American Heart Association’s LE8 score

Domain	CVH Metric	Measurement	Quantification and scoring of CVH Metric
Health behaviors	Diet	Healthy Eating Index-2015 diet score percentile	Scoring (population): Points Quantile 100 ≥95th percentile (top/ideal diet) 80 75th – 94th percentile 50 50th – 74th percentile 25 25th – 49th percentile 0 1st – 24th percentile (bottom/least ideal quartile)
	Physical activity	Self-reported minutes of moderate or vigorous physical activity per week	Metric: Minutes of moderate (or greater) intensity activity per week Scoring: Points (minutes) 100 ≥150 90 120 – 149 80 90 – 119 60 60 – 89 40 30 – 59 20 1 – 29 0 0
	Nicotine exposure	Self-reported use of cigarettes or inhaled nicotine- delivery system	Metric: Combustible tobacco use and/or inhaled NDS use; or secondhand smoke exposure Scoring: Points status 100 Never smoker 75 Former smoker, quit ≥5 yrs 50 Former smoker, quit 1 – <5 yrs 25 Former smoker, quit <1 year, or currently using inhaled NDS 0 Current smoker
			Subtract 20 points (unless score is 0) for living with active indoor smoker in home
	Sleep health	Self-reported average hours of sleep per night	Metric: Average hours of sleep per night Scoring: Points Level 100 7-9 90 9-10
			70	6–7
40	5–6 or ≥10
20	4–5
0	<4
Health Factors	Body mass index	Body weight (kg) divided by height squared (m2)	Metric: Body mass index (kg/m^2^) Scoring: Points level 100 <25 70 25.0 – 29.9 30 30.0 – 34.9 15 35.0 – 39.9 0 ≥40.0
	Blood lipids	Plasma total and HDL-cholesterol with calculation of non-HDL-cholesterol	Metric: Non-HDL cholesterol (mg/dL) Scoring: Points level 100 <130 60 130 – 159 40 160 – 189 20 190 – 219 0 ≥220
			If drug-treated level, subtract 20 points
	Blood glucose	Fasting blood glucose or casual hemoglobin A1c	Metric: Fasting blood glucose (mg/dL) or Hemoglobin A1c (%)
			Scoring: Points level 100 No history of diabetes and FBG <100 (or HbA1c < 5.7) 60 No diabetes and FBG 100 – 125 (or HbA1c 5.7-6.4) (Pre-diabetes) 40 Diabetes with HbA1c <7.0 30 Diabetes with HbA1c 7.0 – 7.9 20 Diabetes with HbA1c 8.0 – 8.9 10 Diabetes with Hb A1c 9.0 – 9.9 0 Diabetes with HbA1c ≥10.0
	Blood pressure	Appropriately measured systolic and diastolic blood pressure	Metric: systolic and diastolic blood pressure (mm Hg) Scoring: Points Level 100 <120/<80 (Optimal)
			75 120-129/<80 (Elevated) 50 130-139 or 80-89 (Stage I HTN) 25 140-159 or 90-99 0 ≥160 or ≥100 Subtract 20 points if treated level


**Table A4:** Association between LC9 and different severity levels of periodontitis

	Crude Model OR (95%CI) p-value	Model 1 OR (95%CI) p-value	Model 2 OR (95%CI) p-value
Periodontitis Normal vs mild
LC9, every 10 points	0.70 (0.66, 0.73) <0.0001	0.73 (0.69, 0.78) <0.0001	0.85 (0.80, 0.90) <0.0001
LC9 quartile			
Q1	Ref.	Ref.	Ref.
Q2	0.66 (0.56, 0.79) <0.0001	0.65 (0.54, 0.78) <0.0001	0.78 (0.64, 0.95) 0.0176
Q3	0.47 (0.39, 0.56) <0.0001	0.47 (0.39, 0.58) <0.0001	0.64 (0.52, 0.79) 0.0003
Q4	0.30 (0.24, 0.38) <0.0001	0.37 (0.29, 0.46) <0.0001	0.62 (0.49, 0.78) 0.0003
p for trend	<0.0001	<0.0001	0.0001
Periodontitis Normal vs moderate			
LC9, every 10 points	0.72 (0.68, 0.76) <0.0001	0.76 (0.72, 0.80) <0.0001	0.87 (0.82, 0.92) 0.0001
LC9 quartile			
Q1	Ref.	Ref.	Ref.
Q2	0.62 (0.52, 0.74) <0.0001	0.61 (0.51, 0.73) <0.0001	0.73 (0.61, 0.88) 0.0024
Q3	0.47 (0.40, 0.56) <0.0001	0.48 (0.40, 0.59) <0.0001	0.64 (0.53, 0.78) 0.0001
Q4	0.33 (0.27, 0.42) <0.0001	0.41 (0.32, 0.52) <0.0001	0.66 (0.52, 0.84) 0.0024
p for trend	<0.0001	<0.0001	0.0017
Periodontitis Normal vs severe			
LC9, every 10 points	0.60 (0.56, 0.64) <0.0001	0.62 (0.57, 0.68) <0.0001	0.75 (0.68, 0.82) <0.0001
LC9 quartile			
Q1	Ref.	Ref.	Ref.
Q2	0.76 (0.58, 0.99) 0.0495	0.76 (0.56, 1.03) 0.0834	0.98 (0.73, 1.31) 0.8898
Q3	0.39 (0.28, 0.53) <0.0001	0.40 (0.28, 0.57) <0.0001	0.61 (0.42, 0.88) 0.0140
Q4	0.13 (0.09, 0.19) <0.0001	0.18 (0.12, 0.27) <0.0001	0.37 (0.26, 0.52) <0.0001
p for trend	<0.0001	<0.0001	<0.0001


**Table A3:** Mediating role of GGT in the association of LC9 with periodontitis

GGT	Estimate	95% CI lower	95% CI upper	p-value
Total effect	-0.053	-0.069	-0.037	<0.001
Mediation effect	-0.001	-0.004	0.001	0.250
Direct effect	-0.052	-0.067	-0.035	<0.001
Proportion mediated	0.022	-0.016	0.071	0.250


**Table A2:** Mediating role of CRP in the association of LC9 with periodontitis

CRP	Estimate	95% CI lower	95% CI upper	p-value
Total effect	-0.045	-0.073	-0.020	<0.001
Mediation effect	-0.004	-0.010	0.001	0.118
Direct effect	-0.042	-0.069	-0.015	0.004
Proportion mediated	0.080	-0.016	0.297	0.118


**Table A5:** Comparative analysis of baseline characteristics of excluded and included participants

	Excluded	Included	p
Age	51.30 ± 0.26	51.33 ± 0.25	0.923
PIR	3.26 ± 0.05	3.26 ± 0.06	0.968
HEI-2015 diet score	44.00 ± 0.56	44.04 ± 0.56	0.957
Physical activity score	73.91 ± 0.80	73.95 ± 0.79	0.952
Nicotine exposure score	74.10 ± 0.64	74.11 ± 0.64	0.993
Sleep health score	83.71 ± 0.42	83.71 ± 0.41	0.986
Body mass index score	59.64 ± 0.60	59.64 ± 0.59	0.998
Blood lipids score	60.79 ± 0.51	60.78 ± 0.50	0.997
Blood glucose score	85.00 ± 0.40	84.98 ± 0.40	0.985
Blood pressure score	68.64 ± 0.58	68.64 ± 0.58	0.996
PHQ9 score	92.07 ± 0.34	92.08 ± 0.34	0.984
LC9	71.32 ± 0.30	71.33 ± 0.30	0.989
Sex			0.12
Male	3570 (49.65)	3579 (49.77)	
Female	3621 (50.35)	3612 (50.23)	
Race			0.089
Mexican American	965 (7.18)	956 (7.09)	
Non-Hispanic Black	1415 (9.50)	1401 (9.43)	
Non-Hispanic White	3445 (72.80)	3458 (73.02)	
Other Hispanic	658 (4.62)	652 (4.57)	
Other Race	730 (5.95)	724 (5.89)	
Marital Status			0.156
Non-single	4700 (70.00)	4725 (70.43)	
Single	2491 (30.00)	2466 (29.57)	
Education			0.112
<Highschool	540 (3.80)	533 (3.76)	
Highschool	2465 (30.05)	2456 (29.94)	
>Highschool	4195 (66.15)	4202 (66.30)	
Drinking alcohol			0.095
Never	885 (9.53)	879 (9.47)	
Former	1255 (14.68)	1251 (14.63)	
Mild	2655 (40.10)	2664 (40.21)	
Moderate	1125 (18.10)	1131 (18.20)	
Heavy	1275 (17.55)	1266 (17.49)	
CVD			0.138
No	6570 (92.50)	6585 (92.74)	
Yes	615 (7.49)	606 (7.26)	
Periodontitis			0.167
No	3640 (59.10)	3651 (59.30)	
Mild	335 (4.24)	337 (4.27)	
Moderate	2495 (29.25)	2490 (29.21)	
Heavy	710 (7.20)	713 (7.24)	
Periodontitis			0.142
No	3640 (59.10)	3651 (59.29)	
Yes	3545 (40.90)	3540 (40.71)	


**Table A7:** Multivariable logistic regression analysis adjusting for additional dental-related variables but not for CVD

Periodontitis	Model 3 OR (95%CI) p-value
LC9, every 10 points	0.86 (0.81, 0.91) <0.0001
LC9 quartile	
Q1	Ref.
Q2	0.79 (0.65, 0.95) 0.0190
Q3	0.66 (0.54, 0.81) 0.0004
Q4	0.65 (0.51, 0.82) 0.0010
p for trend	0.0002
Adjusted for age, sex, race, PIR, marital status, educational level, drinking, bone loss around teeth, floss and mouthwash use.

**Table A6:** Analysis of multicollinearity among mediator variables and adjusted variables

	VIF
Age	1.3
Sex	1.4
Race	1.1
Marital	1.1
PIR	1.4
Education	1.4
Drinking	1.1
CVD	1.1
WBC	4.8
Neutrophil count	4.4
SII	2.4
ALB	1.3
GGT	1.1
UA	1.4
CRP	1.2

